# Morphological classification of the plantaris muscle origin: a cadaveric study

**DOI:** 10.1038/s41598-025-18762-9

**Published:** 2025-11-04

**Authors:** Hyemin Lee, Yijin Heo, Dasom Kim, Seung-jun Hwang

**Affiliations:** 1 Department of Anatomy, College of Medicine, University of Ulsan, 88, Olympic-Ro 43-Gil, Songpa-Gu, Seoul, Korea; 2 Asan Medical Center, Clinical Anatomy Education Center, Seoul, Korea; 3 Department of Anatomy, College of Medicine, Korea University, Seoul, Korea

**Keywords:** Anatomical variation, Cadaver, Plantaris muscle, Morphometry, Republic of Korea, Tennis leg, Anatomy, Medical research

## Abstract

The plantaris muscle (PM), considered vestigial in humans, has clinical significance owing to its anatomical variations. This study examined the prevalence, origin types, and clinical relevance of the PM in the Korean population to aid clinical and surgical practices, particularly in posterior knee surgery. A total of 160 lower limbs (90 male, 70 female) were dissected using classical anatomical methods. The morphology of PM origin was classified, and its prevalence was evaluated. The PM was present in 146 lower limbs (91.25%). Three distinct origin types were observed: Type 1 (68.18%), originating from the knee joint capsule, the lateral condyle of the femur (LFC), and the lateral head of the gastrocnemius muscle (lhGM), with fibers blending into the lhGM tendon; Type 2 (17.53%), originating from the knee joint capsule and the LFC but not blending with the lhGM tendon; and Type 3 (5.19%), originating solely from the LFC and the knee joint capsule, without any attachment to or connection with the lhGM. The average PM muscle belly length and width were 9.56 ± 1.71 cm, and 1.80 ± 0.64 cm, respectively. The PM shows morphological variability according to its origin attachment types. These findings provide valuable baseline data for understanding the anatomy of the PM, which can assist surgeons in diagnosing and treating conditions associated with posterior knee pain.

**Supplementary Information:**

The online version contains supplementary material available at 10.1038/s41598-025-18762-9.

## Introduction

The plantaris muscle (PM), characterized by a short muscle belly and a long tendon, is located in the posterior compartment of the leg. It typically originates from the lateral supracondylar ridge of the femur, above the lateral head of the gastrocnemius muscle and the knee joint capsule. Several reports have documented its morphological variability, including variations in origin, insertion, course, and even absence ^1–4^. Some studies have described the PM as having two or more muscle bellies or tendons ^4–7^.

Herzog et al. ^8^ conducted a retrospective review of 1000 consecutive MRI scans of the knee performed on patients presenting with acute or chronic knee symptoms. They found that 6.3% of patients with posterior knee pain had an accessory PM ^8^. Previous studies have mainly focused on the morphological variations related to the insertion of the PM tendon. Aberrant PM insertion has been identified as a potential trigger for mid-portion Achilles tendinopathy[^13‚17^]. In 2011, Alfredson et al. ^9^ examined 73 consecutive cases of chronic painful mid-portion Achilles tendinopathy and found that an enlarged PM tendon located close to the medial Achilles in 80% of cases, suggesting it as a common factor in patients with chronic painful mid-portion tendinosis.

Owing to the highly variable morphology of the PM, there is a clinical need for an accurate understanding of its anatomical variability. However, compared to studies on PM insertion, research on the variability of the PM origin is limited. The PM is also prone to various injuries, with both the muscle belly and the tendon potentially rupturing at the musculotendinous junction. Such injuries may occur either in isolation or simultaneously with injuries to the gastrocnemius and soleus muscles, a condition referred to as “tennis leg,” initially reported as a clinical condition by Powell in 1883[^20^].

This study aimed to characterize the morphological features of the PM origin and obtain anatomical and physical anthropologic baseline data of the PM. This information has clinical and surgical applications when planning procedures involving this region.

## Materials and methods

### Materials

This study used 160 cadaveric lower limbs (from 90 male and 70 female cadavers) obtained from the anatomy departments of four different medical schools in Korea, including 80 right and 80 left lower limbs. All cadavers were donated to the universities and had been previously used for the education of medical students and clinical fellows. Lower limbs with surgical alterations in the dissection area were excluded from the study. The mean age at death was 80.79 ± 10.02 (range, 54–98) years.

### Methods

#### Dissection & classification of the PM origin

Dissections were performed using traditional dissection techniques. The skin and muscle fascia from the distal half of the femur to the proximal two-thirds of the tibia were removed. The medial head of the gastrocnemius (mhGM) and the lateral head of the gastrocnemius (lhGM) were then identified, with extreme caution exercised to avoid damaging the PM, which lies in close contact proximity to the lhGM. After partially removing the mhGM, the lhGM was transected approximately 5 cm distal to its origin. Once the proximal parts of the PM and soleus muscle (SM) were exposed, unnecessary surrounding tissues were removed to ensure a clear view of the PM. The anatomical structures surrounding the PM were then cleaned to facilitate detailed observation. The presence or absence of the PM and the type of its origin were documented, and morphometric measurements were recorded.

The origin of the PM was classified into three distinct types based on its proximal attachment and anatomical relationship with adjacent structures. The type 1, originated from the lateral supracondylar ridge, lateral condyle of the femur (LFC), and knee joint capsule, with a clear attachment to the lateral head of the gastronemius muscle (lhGM); PM fibers blended into the lhGM tendon. Type 2, shared the same proximal attachment site as type 1, but without blending into the lhGM tendon. And type 3, originated solely from the popliteal surface, the LFC and the knee joint capsule, without any attachment or connection to the lhGM.

#### Measurements

The maximal length and width of the PM muscle belly, along with the width of the myotendinous junction (MTJ), were measured using an electronic digital caliper (CD-15APX, Mitutoyo Corp., Japan). A tape measure with a capacity of up to 100.0 cm was used to determine the tibia length. To measure the tibia length, the practitioner palpated and marked the apexes of the medial tibia condyle and the medial malleolus, and then measured the distance between these two points. All measurements were performed by a single independent practitioner.

#### Statistical analysis

Statistical analysis included morphometric measurements of 140 lower limbs, with 20 cases excluded due to the absence or damage of the PM during dissection. The analyses were performed using IBM SPSS Statistics 29.0 (IBM Corp., Armonk, NY, USA).

Fisher’s exact test was used to analyze the frequency of PM occurrence and to evaluate the association between PM origin types and either gender or body side (Table 1). The normality of continuous variables was assessed using the Shapiro–Wilk test, and homogeneity of variances was verified when appropriate. For comparisons morphometric measurements between genders and body sides (Table 2), independent sample t-tests were used when data met parametric assumptions. When the data were not normally distributed, the Mann–Whitney U test was used to compare anthropometric measurements between genders and body sides.

For comparisons of morphometric measurements among the three PM origin types (Table 3), the Kruskal–Wallis test was used when data were not normally distributed, followed by Dunn’s post-hoc test with Bonferroni correction. When the data were normally distributed but violated the assumption of homogeneity of variances, Welch’s ANOVA was applied, followed by Games-Howell post-hoc test. Unless otherwise stated, data are presented as the mean ± standard deviation (SD), and a  *p-*value < 0.05 was considered statistically significant.

## Results

### Frequency of occurrence of PM and its origin type classification

Out of 160 lower limbs examined, the PM was present in 146 limbs (91.25%) and absent in 14 limbs (8.75%). There was no significant difference in the frequency of occurrence between genders (*P* = 0.801) or body sides (*P* = 0.800) (Tables 1 and 2).

**Table 1:** Classification and of the plantaris muscle origin frequency according to body side and gender difference.

Prevalence of the PM	Gender		*p-*value	Body sides		*p-*value	Total
	M	F		Left	Right		
Age	80.79 ± 10.02						
PM exist	80 (90.91%)	66 (91.67%)	0.801	73 (90.12%)	73 (92.41%)	0.800	146 (91.25%)
PM absence	8 (0.10%)	6 (8.33%)		8 (9.88%)	6 (7.59%)		14 (8.75%)
Total	88	72		81	79		160*

*90 male and 70 female cadavers obtained from these results.

**Table 2:** Morphometric measurements of the plantaris muscle according to body side.

Features	Left	Right	*P*-value	Total
Age	80.79 ± 10.02			
BL	9.45 ± 1.74	9.71 ± 1.67	0.512	9.56 ± 1.71
BW	1.77 ± 0.57	1.81 ± 0.70	0.861	1.80 ± 0.64
MTJW	0.33 ± 0.12	0.33 ± 0.11	0.903	0.33 ± 0.12
TL	30.84 ± 1.84	30.75 ± 1.78	0.910	30.78 ± 1.81
BL/TL	0.31 ± 0.06	0.32 ± 0.05	0.553	0.32 ± 0.06
Total	70	70		140*

*The total of 140 lower limbs represents the cases remaining after excluding 14 cases of PM absence and 6 cases of damaged PM origin during the examination of 160 lower limbs.

BL, muscle belly length; BW, muscle belly width; MTJW, myotendinous junction width; TL, tibia length.

The classification was based on the exact location of the origin, the attachment of the proximal muscle belly, and the course of the muscle belly in the posterior knee compartment. Type 1 was the most frequent origin (68.18%), followed by Type 2 (17.53%) and Type 3 (5.19%). No significant differences were observed in the distribution of the origin types between genders (*P* = *0.072*) body sides (*P* = *0.314*) (Table 3).

**Table 3:** Classification of plantaris muscle origin types by gender and body side.

Type of origin	Gender		*P*-value	Body side		*P*-value	Total
	Male	Female		Left	Right		
1	65 (73.86%)	40 (60.61%)	0.072	56 (71.79%)	49 (64.47%)	0.314	105 (68.18%)
2	11 (12.5%)	16 (24.24%)		10 (12.82%)	17 (18.42%)		27 (17.53%)
3	3 (3.41%)	5 (7.58%)		4 (5.13%)	4 (5.26%)		8 (5.16%)
Absence	9(10.23%)	5 (7.58%)		8 (10.25%)	6 (7.89%)		14 (9.09%)
Total	88	66		78	76		154 *

*The total of 154 lower limbs represents the cases remaining after excluding 6 cases of damaged PM origin during the examination of 160 lower limbs. Null data due to, damage to the PM origin during dissection = 6 cases (2 left lower limbs and 4 right lower limbs).

### Morphological characteristics of the origin of the PM

**Fig. 1:**
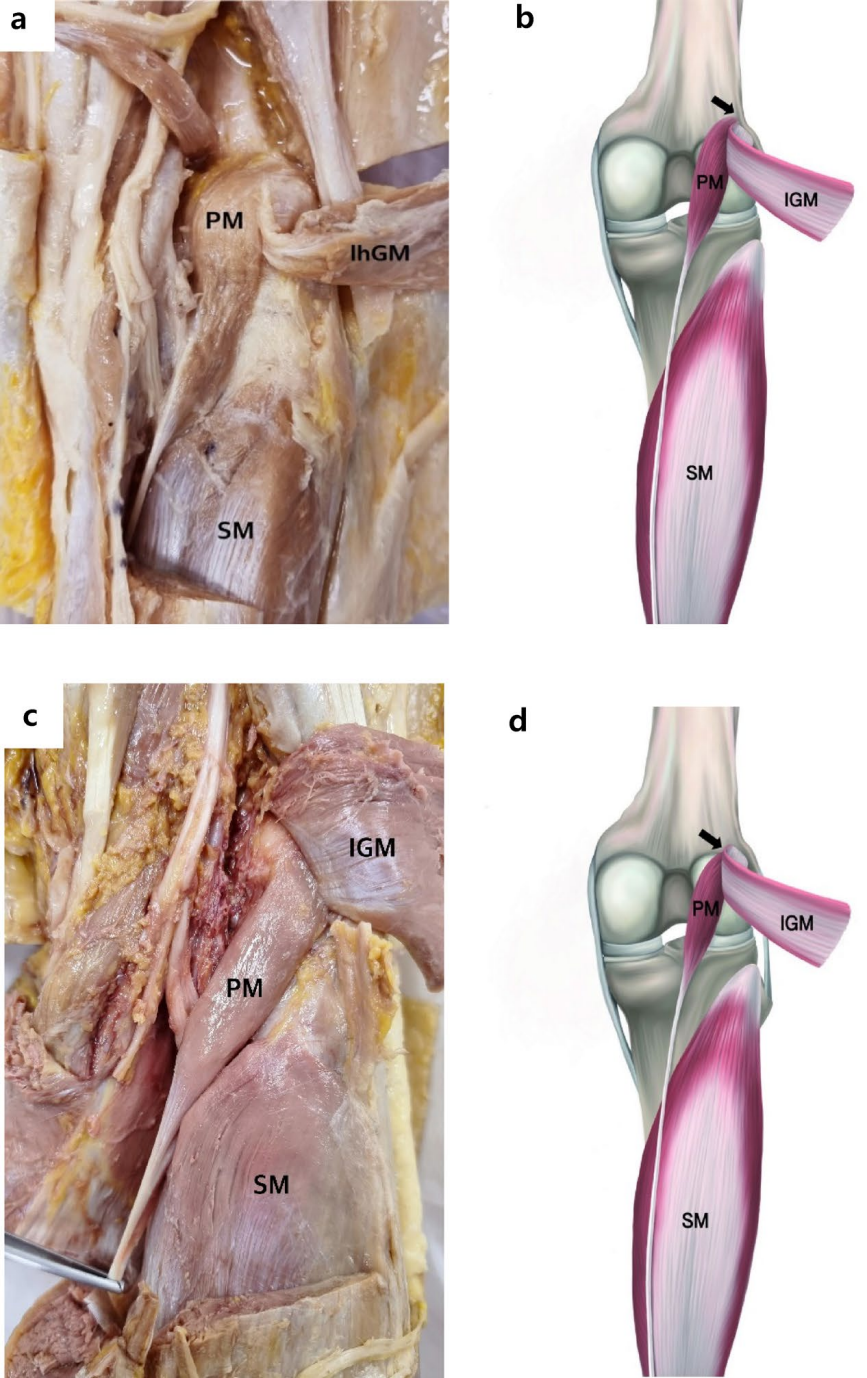

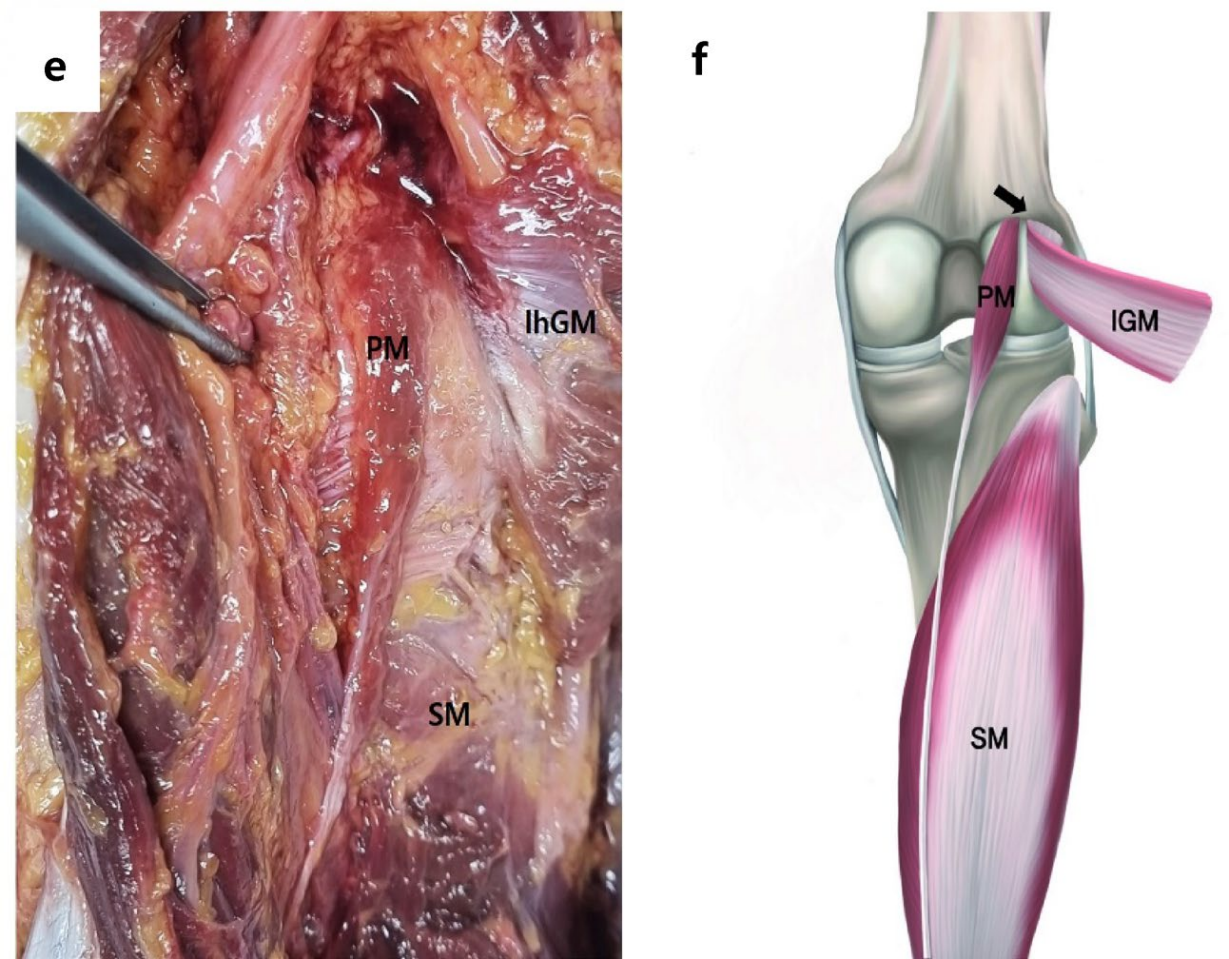
Classification of PM origin types. Photographs of the posterior view of the knee showing the Type 1 origin of the PM (**a**) Type 2 origin (**c**), and Type 3 origin (**e**). Corresponding schematic drawings show Type 1 origin (**b**), Type 2 origin (**d**), and Type 3 origin (**f**). PM; plantaris muscle, lhGM; lateral head of the gastrocnemius muscle, SM; soleus muscle.

The three types of PM origins were classified based on the location of their proximal muscle belly attachment, which involved the posterior knee joint capsule, lateral condyle of the femur, and the lhGM. A photograph of a dissected cadaver is shown in (Fig. 1a, c, e) and a schematic drawing of three types PM origin is shown in (Fig. 1b, d, f). And the detailed descriptions of the PM origin types are as follows:

1. *Type 1* The PM originated from the lateral supracondylar ridge, lateral condyle of the femur (LFC), and the knee joint capsule, with an attachment to the lhGM. The muscle fibers of the PM blend with the lhGM tendon. A representative photograph and its corresponding schematic diagram were shown in Fig. 1a + b.
2. *Type 2* The origin is located at the knee joint capsule and the popliteal surface, the LFC, also with an attachment to the lhGM. However, the PM dose not blend with the lhGM tendon. This type was photograph and its corresponding schematic illustration was shown in Fig. 1c + d.
3. *Type 3* The origin is located solely on the popliteal surface, just above LFC and the knee joint capsule, with no attachment or connection to the lhGM. This type was photographed and its corresponding schematic illustration, which were included in Fig. 1e + f.

### Morphometric measurements of the PM

Excluding cases of PM absence and six damaged PM origin specimens during dissection, we examined a total of 140 PM origins and classified them into three types. The muscle belly length and width, MTJ width of the PM, and the tibia length were measured in 140 lower limbs. The ratio of the PM muscle belly length to tibia length represents the relative location of the MTJ in relation to tibial length. The mean measurements were as follows: muscle belly length, 9.56 ± 1.71 cm; muscle belly width, 1.80 ± 0.64 cm; MTJ width, 0.33 ± 0.12 cm; and tibia length, 30.78 ± 1.81 cm. The ratio of the PM muscle belly length to tibia length was 0.32 ± 0.06.

No significant differences were observed in any measurements between body sides (Table 2). However, significant differences between genders were observed in all measurements, except for the ratio of PM muscle belly length to tibia length (Supplementary Information Table S1).

### Comparisons of PM morphological measurements between origin types

**Fig. 2:**
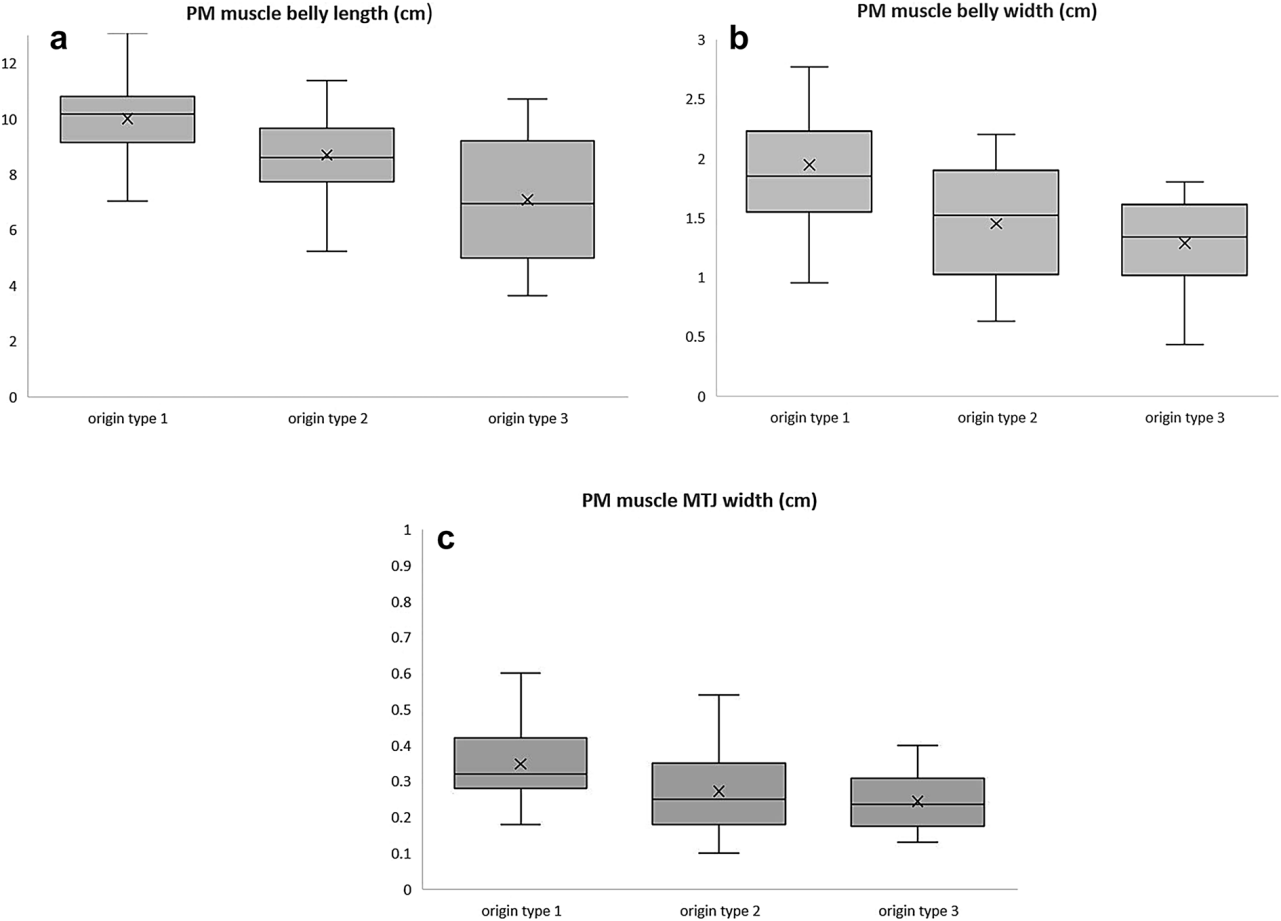
Comparison of PM belly length (**a**), PM belly width (**b**), and PM MTJ width (**c**) across the three PM origin types. Statistically significant differences were observed among the groups (*p* < 0.001, Kruskal–Wallis test). Pairwise comparisons were performed using Dunn’s post-hoc test with Bonferroni correction. *p ≤ 0.05, ** *p* ≤ 0.01, *** *p* ≤ 0.001; ns = not significant (*p* > 0.05; data not shown).

The muscle belly length, muscle belly width, and MTJ width of the PM were compared among all origin types. Boxplots were generated to illustrate the relationships between the origin types (Fig. 2). The mean muscle belly length for PM origin Type 1 was 10.00 ± 1.42 cm, for Type 2 was 8.69 ± 1.53 cm, and for Type 3 was 7.08 ± 2.47 cm. The mean muscle belly width for PM origin Type 1 was 1.94 ± 0.64 cm, that for Type 2 was 1.45 ± 0.50 cm, and that for Type 3 was 1.29 ± 0.44 cm. The mean MTJ width for PM origin Type 1 was 0.35 ± 0.11 cm, that for Type 2 was 0.27 ± 0.12 cm, and that for Type 3 was 0.24 ± 0.09 cm.

All comparisons, of muscle belly length, muscle belly width, and MTJ width among the different PM origin types showed statistically significant differences (*P* < 0.001). In addition, comparisons by gender and body side for all PM origin types showed statistically significant results (*P* < 0.001) (Supplementary Information Figure S1 and S2).

## Discussion

Due to considerable difference of its presence frequency, there has been an ongoing debate about whether the PM is a vestigial muscle. In 2021, Gonera et al. conducted a comprehensive review of the existing literature on the PM. The authors did not consider the PM to be a potential vestigial organ. They suggested if the PM were a vestigial organ, it should show a clear trend of decreasing throughout human evolutionary history. However, their analysis of the prevalence of the PM across three population groups—European, American, and Asian—did not reveal any definitive trend of increasing or decreasing frequency within these groups. Notably, studies conducted after 2000 in American and Asian populations reported a PM prevalence of over 90% in most cases. Consequently, the current data are deemed insufficient to conclusively determine whether the PM is a vestigial organ ^10^. Consistent with these findings, our study found that the prevalence of the PM among Koreans was 91.25%.

The abnormal morphology of the PM has clinical significance, particularly as a potential cause of popliteal artery entrapment syndrome (PAES) ^11^. Kwon et al. confirmed that anatomical abnormalities, such as an aberrant PM or an abnormal mhGM, could contribute to PAES. Their analysis of MRI and CT scans from 35 PAES-affected legs revealed that 26.9% of patients had an aberrant PM, located higher and more medially than the normal course of the PM. This structure can compress the popliteal artery during plantar flexion of the ankle, potentially leading to artery occlusion. These findings highlight the importance of considering the PM’s anatomical characteristics in both comparative anatomy and clinical research, particularly its muscle belly shape and origin.

In our study, we examined 160 lower limbs of Koreans and identified 140 distinct PM origins, which were classified into three types. Among these, Type 1 and Type 2 were the most common. The classification criteria were based on whether the PM originated from the lhGM, the LFC, or the knee joint capsule. These types demonstrate a connection between the PM and the lhGM, which may suggest increased interaction between these two muscles. Notably, the Type 1 PM origin is attached to both the proximal part of the LFC and the knee joint capsule, with muscle fibers blending with the lhGM tendon. This structure may enhance stability and contribute to an increased range of motion during muscle contraction, allowing the PM to function more effectively in stabilizing posterior leg movements ^12‚19^. In contrast, Type 3 PM is attached only to the popliteal surface of the LFC and the knee joint capsule, without any connection to the lhGM.

Several previous studies have categorized PM origin types using their own classification systems ^1,12‚18^. The results of this study align with the classifications proposed by earlier researchers, showing notable similarities. In particular, the morphological characteristics and frequencies of PM origin types observed in Koreans closely resemble those reported by Nayak et al.’s study on the Indian population. When compared to the studies by Freeman et al. and Olewnik et al., which focused on European populations, both Nayak et al.’s findings and those of this study reveal a significantly higher occurrence rate of PM origin Type 1. These results suggest the possibility of racial differences in PM origin types. In terms of specific characteristics, PM origin Type 2 (attached to the LFC, knee joint capsule, and lhGM) was consistently observed by all researchers, while PM origin Types 1 and 3 were reported in only some studies. Additionally, in our study, we did not observe rare cases of PM attachment to the iliotibial band, patella, or fibular collateral ligament, as reported in previous studies (Table 4).

**Table 4:** Comparison of plantaris muscle origin classifications across studies.

PM Origin attachment	Freeman et al*.* (n = 46)	Nayak et al*.* (n = 52)	Olewnik et al*.* (n = 142)	Present study (n = 154)
Attached to lhGM and mixed with lhGM tendon, LFC, KN	–	79.1%	40.4%	68.18%
lhGM, LFC, KN, accessory muscle belly attached to the popliteal surface of the femur	–	–	8.7%	–
Attachment to lhGM and not mixed with lhGM, popliteal surface of the LFC, and KN	22.5%	6.3%	25.4%	17.53%
LFC, KN	65.0%	–	10.4%	5.19%
LFC, KN, iliotibial band	–	–	6.4%	–
Narrow attachment to the LFC	–	–	8.7%	–
Fibrous extension of the PM to the patella	12.5%	–	–	–
lhGM, LFC, KN, fibular collateral ligament	–	14.6%	–	–
Prevalence of the PM	87%	92.3%	90.1%	91.25%

lhGM, lateral head of the gastrocnemius; LFC, lateral condyle of the femur; KN, knee joint capsule.

Examining the distribution of PM origin types revealed that the ranking of each type was consistent between males and females. However, the proportions of Type 2 and Type 3 origins were higher in females compared to males (Table 2). Additionally, measurements of PM muscle belly length, muscle belly width, MTJ width, and tibia length were all significantly greater in males. In contrast, the ratio of PM muscle belly length to tibia length which represents the relative position of the MTJ, did not differ between genders (Supplementary Information Table S1).

From a physical anthropological perspective, gender differences in muscle development are influenced by various factors, including biomechanical demands and hormonal effects ^14^. The greater muscle mass and size observed in males can be attributed to a higher level of testosterone, which promotes muscle hypertrophy. Additionally, males tend to have stronger tendons at muscle attachment points owing to their larger muscle mass and higher biomechanical demands. We propose that these factors contribute to stronger attachment of the PM origin and larger muscle belly size in males, which may explain the higher prevalence of Type 1 PM origin in males compared to females. Further biomechanical and physiological studies are recommended to investigate the underlying mechanisms behind these findings.

To further analyze the relationship between PM morphological characteristics and their origin types, we compared the PM muscle belly length, muscle belly width, and MTJ thicknesses for each type. PM origin Type 1 exhibited a relatively longer muscle belly, greater muscle width, and thicker MTJ compared to other types. Type 2 origins had a smaller muscle belly and thinner MTJ compared to Type 1, while Type 3 showed the smallest muscle belly size and thinnest MTJ width among the three types (Figure 2). These differences suggest that Type 2 and Type 3 origins may be more vulnerable to complex injuries in the posterior leg.

The MTJ plays an important role in force transmission and stabilization and is a known site of injury, particularly in muscles span two joints, such as the GM and PM. Injuries at the posterior knee MTJ have been reported to affect outcomes, especially when not properly addressed ^15,16^. In this study, we identified the relative position of the PM MTJ using the ratio of muscle belly length to tibial length, which was 0.32 ± 0.06. While this finding does not directly indicate clinical utility, it may serve as a reference point for future anatomical or imaging-based studies aiming to understand posterior knee pain or assist in surgical planning.

One limitation of our study is its focus on anatomical variations in the PM origin without assessment of PM insertion characteristics. Nevertheless, the data presented here provide base line anatomical data on the PM in Koreans, contributing to a broader understanding of the morphological characteristics of the PM. Additionally, the estimation of MTJ location in relation to tibial length may support further clinical studies, particularly those addressing knee injuries and surgical treatments.

## Supplementary Information

Below is the link to the electronic supplementary material.

Supplementary Material 1

## Data Availability

The data can be made available as and when requires by the journal. The datasets used during the current study available from the corresponding author on reasonable request. Corresponding author: Seung-Jun Hwang, e-mail: sjhwang@amc.seoul.kr.
