# Supplementary material for: Morphological classification of the plantaris muscle origin: a cadaveric study
Source: Sci Rep. 2025 Nov 4;15:38527. doi: 10.1038/s41598-025-18762-9 (PMC12586595; doi:10.1038/s41598-025-18762-9)
Supplement: Supplementary file 1 — Supplementary Material 1 [file 41598_2025_18762_MOESM1_ESM.docx]

**Supplementary Information (SI)**

Supplementary Table S1. Morphometric measurements of the plantaris muscle according to gender

| **Features** | **Gender** | | | |  | **Total**  **(n = 140)** |
| --- | --- | --- | --- | --- | --- | --- |
|  | **Male** | | | **Female** | *P*-value |  |
| **BL**  **BW**  **MTJW** | |  | 9.99 ± 1.69 | 9.04 ± 1.58 | <0.001*  <0.001*  0.008* | 9.56 ± 1.71 |
|  |  |  | 1.98 ± 0.68 | 1.55 ± 0.48 |  | 1.80 ± 0.64 |
|  |  |  | 0.35 ± 0.12 | 0.30 ± 0.09 |  | 0.33 ± 0.12 |
| **TL** | |  | 31.57 ± 1.46 | 29.79 ± 1.72 | <0.001* | 30.78 ± 1.81 |
| **BL/TL** | |  | 0.32 ± 0.06 | 0.30 ± 0.05 | 0.068 | 0.32 ± 0.06 |
| **Total** | |  | 79 | 61 |  | 140 |

BL; muscle belly length, BW: muscle belly width, MTJW: myotendinous junction width, TL: tibial length

*statistically significant


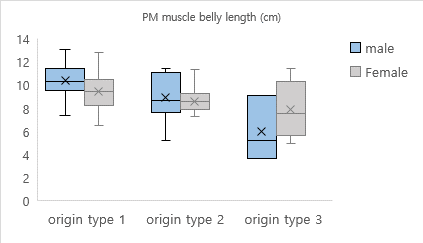

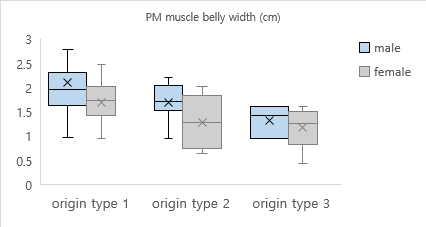


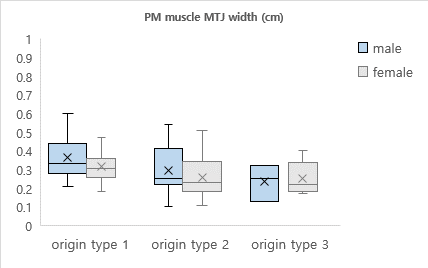


Supplementary Figure S1. Comparison of PM muscle belly length, muscle belly width, and MTJ width between PM origin types and genders


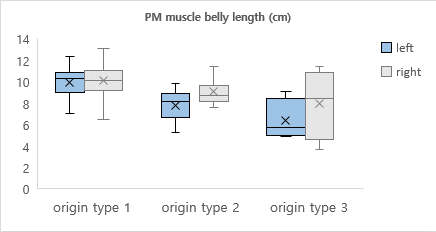


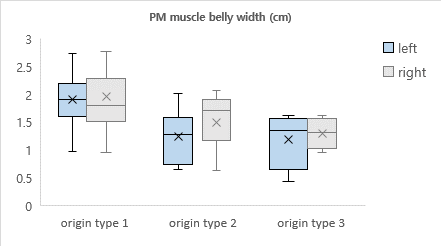


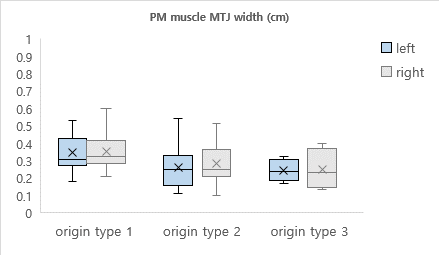


Supplementary Figure S2. Comparison of PM muscle belly length, muscle belly width, and MTJ width between PM origin types and body sides
